# Isolation and Characterization of Three Pseudomonas aeruginosa Viruses with Therapeutic Potential

**DOI:** 10.1128/spectrum.04636-22

**Published:** 2023-05-01

**Authors:** Xiao Wang, Jingjing Tang, Wen Dang, Zhen Xie, Fuhua Zhang, Xinwei Hao, Sihuai Sun, Xuan Liu, Yi Luo, Mengyuan Li, Yanchao Gu, Yao Wang, Qiwei Chen, Xihui Shen, Lei Xu

**Affiliations:** a State Key Laboratory of Crop Stress Biology for Arid Areas, Shaanxi Key Laboratory of Agricultural and Environmental Microbiology, College of Life Sciences, Northwest A&F University, Yangling, Shaanxi, China; b State Key Laboratory of Veterinary Etiological Biology, College of Veterinary Medicine, Lanzhou University, Lanzhou Veterinary Research Institute, Chinese Academy of Agricultural Sciences, Lanzhou, China; University of California, San Diego

**Keywords:** *Pseudomonas aeruginosa*, bacteriophage, phage therapy, biofilm, burst size

## Abstract

As one of the most common pathogens of opportunistic and hospital-acquired infections, Pseudomonas aeruginosa is associated with resistance to diverse antibiotics, which represents a significant challenge to current treatment modalities. Phage therapy is considered a promising alternative to conventional antimicrobials. The characterization and isolation of new bacteriophages and the concurrent evaluation of their therapeutic potential are fundamental for phage therapy. In this study, we employed an enrichment method and a double-layer agar overlay to isolate bacteriophages that infect P. aeruginosa strains PAO1 and PA14. Three phages (named PA_LZ01, PA_LZ02, and PA_LZ03) were isolated and showed icosahedral heads and contractile tails. Following full-genome sequencing, we found that phage PA_LZ01 contained a genome of 65,367 bp in size and harbored 90 predicted open reading frames (ORFs), phage PA_LZ02 contained a genome of 57,243 bp in size and harbored 75 predicted ORFs, and phage PA_LZ03 contained a genome of 57,367 bp in size and carried 77 predicted ORFs. Further comparative analysis showed that phage PA_LZ01 belonged to the genus *Pbunavirus* genus, phage PA_LZ02 belonged to the genus *Pamexvirus*, and phage PA_LZ03 belonged to the family *Mesyanzhinovviridae*. Next, we demonstrated that these phages were rather stable at different temperatures and pHs. One-step growth curves showed that the burst size of PA_LZ01 was 15 PFU/infected cell, and that of PA_LZ02 was 50 PFU/infected cell, while the titer of PA_LZ03 was not elevated. Similarly, the biofilm clearance capacities of PA_LZ01 and PA_LZ02 were also higher than that of PA_LZ03. Therapeutically, PA_LZ01 and PA_LZ02 treatment led to decreased bacterial loads and inflammatory responses in a mouse model. In conclusion, we isolated three phages that can infect P. aeruginosa, which were stable in different environments and could reduce bacterial biofilms, suggesting their potential as promising candidates to treat P. aeruginosa infections.

**IMPORTANCE** Phage therapy is a promising therapeutic option for treating bacterial infections that do not respond to common antimicrobial treatments. Biofilm-mediated infections are particularly difficult to treat with traditional antibiotics, and the emergence of antibiotic-resistant strains has further complicated the situation. Pseudomonas aeruginosa is a bacterial pathogen that causes chronic infections and is highly resistant to many antibiotics. The library of phages that target P. aeruginosa is expanding, and the isolation of new bacteriophages is constantly required. In this study, three bacteriophages that could infect P. aeruginosa were isolated, and their biological characteristics were investigated. In particular, the isolated phages are capable of reducing biofilms formed by P. aeruginosa. Further analysis indicates that treatment with PA_LZ01 and PA_LZ02 phages reduces bacterial loads and inflammatory responses *in vivo*. This study isolated and characterized bacteriophages that could infect P. aeruginosa, which offers a resource for phage therapy.

## INTRODUCTION

Phages are abundant in the natural environment, and the number is estimated to reach 10^31^ to 10^32^, which is 10 times the number of bacteria (1). These viruses have been of great interest since Frederick Twort first observed a “glassy transformation” in colonies in 1915, and phage therapy was proposed as a treatment for bacterial infections at that time (2). However, the widespread use of antibiotics overshadowed the potential of phage therapy. With the emergence of antibiotic resistance, the development of phage treatment has regained attention (3). Phages offer several advantages over antibiotics, including their specificity for bacteria and their ability to evolve rapidly to target new strains of bacteria (4). These unique features have sparked interest in phage therapy as a potential alternative to antibiotics in the fight against bacterial infections, particularly multidrug-resistant bacteria. Although safety and efficacy concerns have been raised, phage therapy is a viable treatment option for overcoming the growing threat of antibiotic resistance, and the number of clinical trials using phages registered at ClinicalTrials.gov (https://clinicaltrials.gov/ct2/home) has increased (5).

Pseudomonas aeruginosa is a common Gram-negative opportunistic bacterium that is widespread in different environments (6). It causes hospital-acquired and pulmonary infections and can form biofilms during chronic infection, which confers resistance to antibiotics, host cells, and mechanical clearance (7). P. aeruginosa can cause fatal infections in patients with cancer, surgery, severe burns, AIDS, or cystic fibrosis (8–10). In cystic fibrosis patients, P. aeruginosa can persistently infect the lungs, resulting in recurrent chronic lung infections (10).

Currently, the treatment of P. aeruginosa infection relies mainly on various antibiotics. However, the emergence of multidrug-resistant P. aeruginosa hampers the clearance of P. aeruginosa infection, and new antimicrobial strategies are required. Phage therapy has been considered an alternative to traditional antibiotics for the treatment of bacterial infections, including those caused by multidrug-resistant P. aeruginosa strains (11–15). Unlike antibiotics, which typically target a broad range of bacteria, bacteriophages are highly specific for their target bacterial species or even strains. This specificity minimizes the impact of phage therapy on the normal microbial flora, reducing the risk of dysbiosis and secondary infections (16, 17). In addition to their high specificity, bacteriophages can also self-propagate, which means that they can replicate and amplify within the host until the infection is cleared. This self-replication property allows lower dosages and shorter treatment durations, thus reducing the risk of side effects and the development of drug resistance (17, 18).

One important feature of P. aeruginosa is its ability to form biofilms on various surfaces, including medical devices, wounds, and lung tissues in patients with cystic fibrosis (7, 19). Biofilms provide a protective environment for bacteria, making them resistant to antibiotics and immune responses (20, 21). In addition to its potential to lyse bacterial cells, phage infection also has the ability to reduce the formation of P. aeruginosa biofilms (22). Together, the potential benefits of phage therapy for the treatment of P. aeruginosa infections make it an attractive alternative to traditional antibiotics.

In this study, we isolated and characterized three P. aeruginosa phages (PA_LZ01, PA_LZ02, and PA_LZ03) and investigated their phagic properties, including their morphology, stability, lytic properties, and effectiveness in clearing biofilms. Additionally, we sequenced and analyzed the genomes of these phages. To evaluate their potential for clinical application, we treated P. aeruginosa-infected mice with phages PA_LZ01 and PA_LZ02. Our findings provide a source for the future development and application of phage therapy.

## RESULTS

### Isolation and characterization of the phages that infect P. aeruginosa.

**FIG 1:**
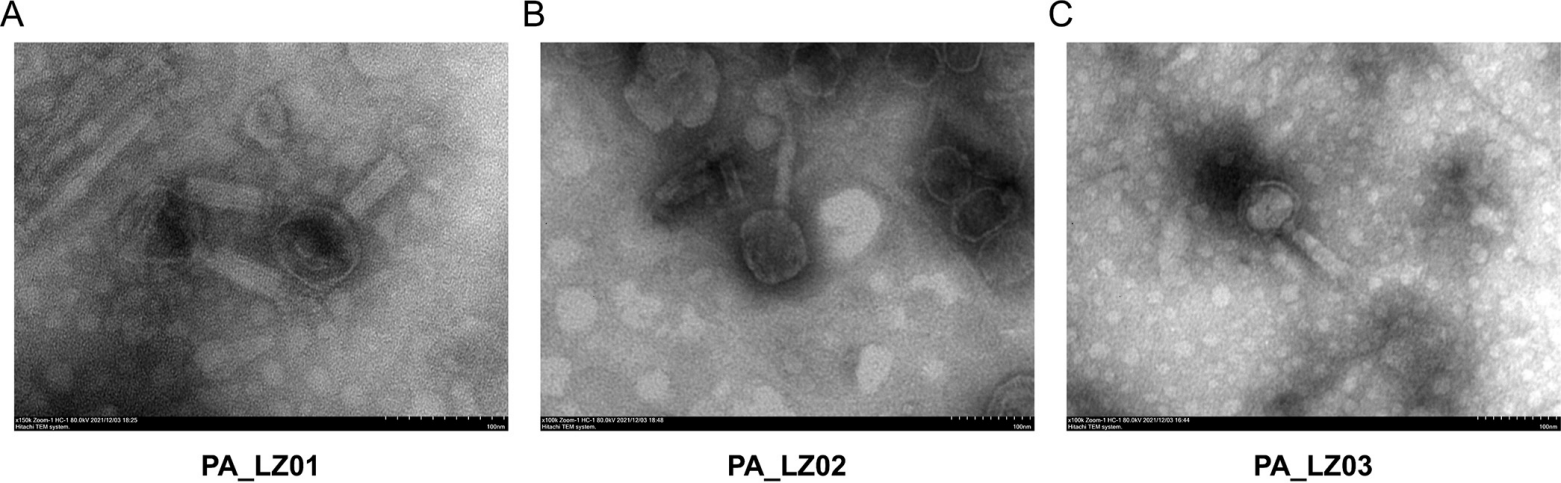
Transmission electron microscopy images of phages PA_LZ01 (A), PA_LZ02 (B), and PA_LZ03 (C) that were negatively stained with 2% phosphotungstic acid.

To isolate bacteriophages that infect P. aeruginosa, wastewater from a hospital sewer was collected to infect two P. aeruginosa strains, PAO1 and PA14 (23). Two bacteriophages, named PA_LZ01 and PA_LZ02, that can infect PAO1 were isolated, and one bacteriophage, named PA_LZ03, was able to infect strains PAO1 and PA14 (see Table S3 in the supplemental material). All three bacteriophages formed visible plaques in a double-layer agar overlay plate and the plaques were round and pure (Fig. S1A to C) (24). The structures of these bacteriophages were observed by using electron microscopy, and all phages had icosahedral heads and contractile tails (Fig. 1A to C). PA_LZ01 had a capsid structure size of 62 ± 4 nm in diameter and a tail length of 71 ± 3 nm (Fig. 1A). The capsid structure sizes of PA_LZ02 and PA_LZ03 were 90 ± 3 nm and 70 ± 3 nm in diameter, respectively, and their tail lengths were both 91 ± 2 nm (Fig. 1B and C). All genomes were prone to degradation by DNase I but not RNase A, indicating that their genetic material was DNA (Fig. S2).

### Basic characteristics and sequencing of the genomes.

**FIG 2:**
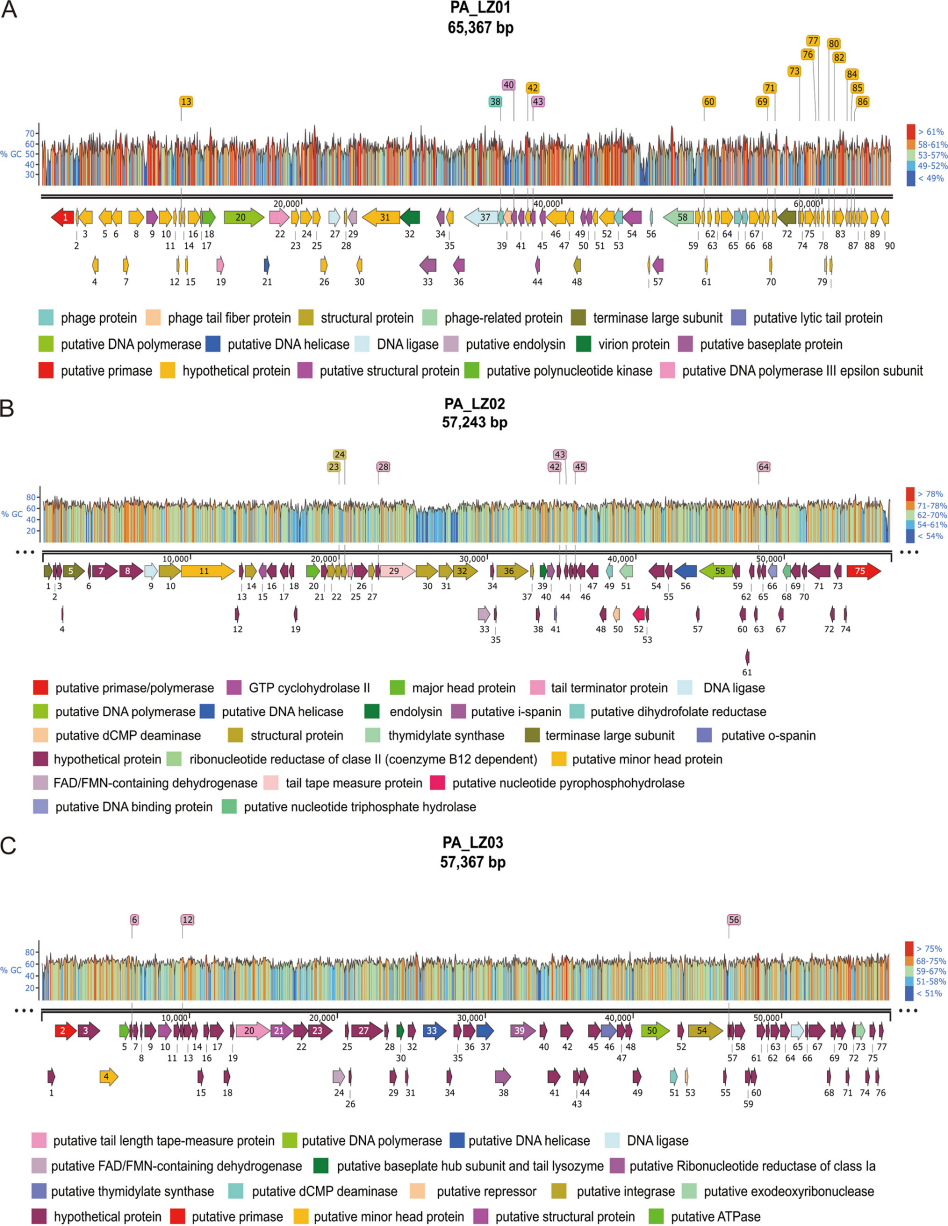
Genome maps of phages PA_LZ01 (A), PA_LZ02 (B), and PA_LZ03 (C). The arrows indicate the predicted open reading frames, and the different colors indicate the diverse functions of the encoded proteins.

To further explore the genomic sequences of the three phages, their genomes were paired-end (PE) sequenced using Illumina sequencing technology. The effective reads were assembled, the contigs were cyclically filled in the boundary in the SPAdes software environment until they could not be extended, and complete scaffolds were generated. The length of the PA_LZ01 genome is 65,367 bp, and it harbors approximately 91.35% coding regions with 90 predicted open reading frames (ORFs) as well as a 55.61% G+C content (Table S4). Of note, we did not find integrase, excisionase, and repressor genes in the genome of PA_LZ01, which are considered indicative of the potential for a lysogenic cycle. These data suggested that phage PA_LZ01 is a virulent phage. Based on functional annotations, predicted phage proteins were grouped into the following categories: host lysis (including a putative endolysin, ORF29), DNA packaging (including a terminase large subunit, ORF72), DNA replication and repair (including a putative DNA polymerase III epsilon subunit, ORF19; a putative DNA polymerase, ORF20; putative DNA helicases, ORF21 and -22; and a DNA ligase, ORF27), viral structural proteins (including putative baseplate proteins, ORF33 and -34; a putative lytic tail protein, ORF37; a phage tail fiber protein, ORF39; and a structural protein, ORF48), nucleotide metabolism (including a putative polynucleotide kinase, ORF18), and hypothetical proteins (Fig. 2A). The genome of phage PA_LZ02 is 57,243 bp in length, with a 66% G+C content and 75 predicted ORFs. The genomic analysis did not identify temperate phage marks such as integrase, excisionase, and repressor genes. This suggests that phage PA_LZ02 is also a virulent phage. Based on functional annotations, predicted phage proteins were grouped into the following categories: host lysis (including a putative endolysin, ORF39), DNA packaging (including terminase large subunits, ORF1 and -5), DNA replication and repair (including a DNA ligase, ORF9; a DNA helicase, ORF56; a DNA polymerase, ORF58; and a putative primase/polymerase, ORF75), viral structural proteins (including structural proteins, ORF14, -22 to -24, -30 to -32, -36, and -37; head proteins, ORF11 and -20; a tail terminator protein, ORF25; a major tail structural protein, ORF26; and a tail tape measure protein, ORF29), nucleotide metabolism (including a ribonucleotide reductase of class II [coenzyme B_12_ dependent], ORF7; a GTP cyclohydrolase II, ORF15; a flavin adenine dinucleotide [FAD]/flavin mononucleotide [FMN]-containing dehydrogenase, ORF33; a putative dihydrofolate reductase, ORF49; a putative dCMP deaminase, ORF50; a thymidylate synthase, ORF51; a putative nucleotide pyrophosphohydrolase, ORF52; and a putative nucleotide triphosphate hydrolase, ORF62), phage lysis proteins (including a putative i-spanin, ORF41, and a putative o-spanin, ORF42), and hypothetical proteins (Fig. 2B). The genome of phage PA_LZ03 is 57,367 bp in length, with 63.4% G+C content and 75 predicted proteins. We found an integrase (ORF53) and a repressor (ORF54) in the genome of PA_LZ03, which are the hallmarks of a temperate phage. Therefore, phage PA_LZ03 might be a temperate phage. Based on functional annotations, predicted phage proteins were grouped into the following categories: DNA packaging (including a terminase large subunit, ORF2), DNA replication and repair (including a DNA helicase, ORF33; a putative RecD-like DNA helicase, ORF37; a putative DNA polymerase I, ORF50; and a putative DNA ligase, ORF65), viral structural proteins (including a putative minor head protein, ORF4; a putative tail length tape measure protein, ORF20; a structural phage protein, ORF21; and a putative baseplate hub subunit and tail lysozyme, ORF30), nucleotide metabolism (including a putative ATPase, ORF5; a putative FAD/FMN-containing dehydrogenase, ORF24; putative ribonucleotide reductases of class Ia, ORF38 and -39; a putative thymidylate synthase, ORF46; a putative dCMP deaminase, ORF51; and a putative exodeoxyribonuclease, ORF73), and hypothetical proteins (Fig. 2C).

### Comparative genome analysis.

**FIG 3:**
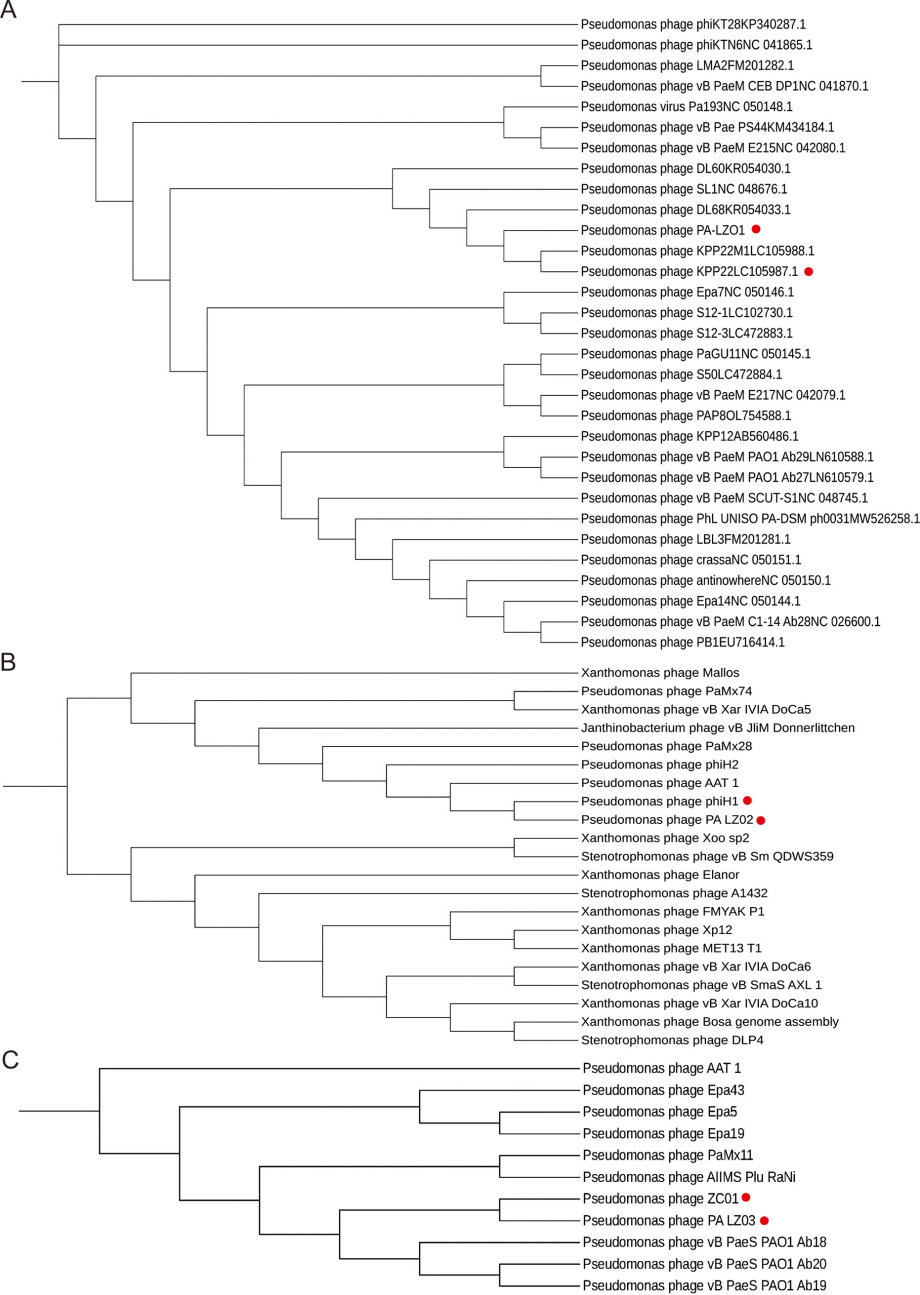
Phylogenetic relationships among phages. (A) Maximum likelihood tree showing the phylogenetic relationships among phages of the genus *Pbunavirus*. Phage PA_LZ01 and phage KPP22 are marked with a red dot. (B) Maximum likelihood tree showing the phylogenetic relationships among phages of the genus *Pamexvirus*. Phage PA_LZ02 and phage phiH1 are marked with a red dot. (C) Maximum likelihood tree showing the phylogenetic relationships among phages of the family *Mesyanzhinovviridae*. Phage PA_LZ03 and phage ZC01 are marked with a red dot. The whole-genome sequences were aligned by using MAFFT, and the tree was visualized by using MEGA 7. The values at the nodes indicate the bootstrap support scores as calculated using 1,000 replicates.

**FIG 4:**
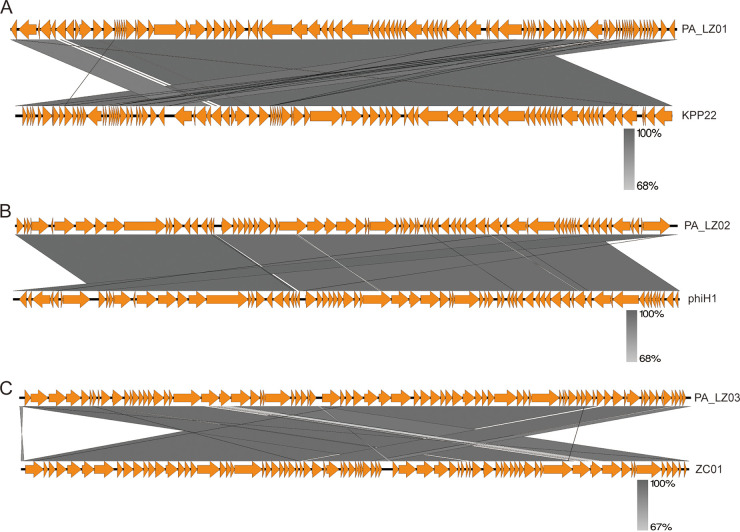
Genomic comparison of the three phages. (A) Genomic organizations of phage PA_LZ01 and phage KPP22. (B) Genomic organizations of phage PA_LZ02 and phage phiH1. (C) Genomic organizations of phage PA_LZ03 and phage ZC01. Each arrow represents an ORF. The different shades of gray represent different identity levels. The figure was created using Easyfig.

Based on the sequencing results, similar sequences were retrieved from the NCBI database for homology analysis of different phages (Table S5). The BLASTN results indicated that PA_LZ01 belonged to the genus *Pbunavirus* of the class *Caudoviricetes*, which is relatively common for Pseudomonas phages (Fig. 3A). The BLASTN results indicated that PA_LZ02 belonged to the genus *Pamexvirus* and that PA_LZ03 belonged to the family *Mesyanzhinovviridae* (Fig. 3B and C). Generally, the genomes of viruses of the genus *Pbunavirus* range from 64.1 to 68.9 kb, and the G+C contents range from 54.9% to 55.8%, with 86 to 95 ORFs, which is in good agreement with our sequencing results. Viruses of the genus *Pbunavirus* lack RNA polymerase and tRNA genes, suggesting that phages of this genus are completely dependent on the host’s transcription machinery. Next, we used MAFFT for sequence analysis and MEGA for visualization of the results. PA_LZ01 showed close relationships to phage KPP22 (GenBank accession number LC105987.1) and KPP22M1 (accession number LC105988.1) and distant relationships to other phages, which is indicative of the low level of homology of PA_LZ01 to other *Caudoviricetes*. By comparing the core genes, PA_LZ01 and phage KPP22 (GenBank accession number LC105987.1) were more closely related (Fig. 4A). Together, we found that PA_LZ01 belongs to the genus *Pbunavirus* and has a high level of similarity with phage KPP22 (GenBank accession number LC105987.1). We further showed that PA_LZ02 exhibited a close relationship to phage phiH1 (Fig. 4B) and that PA_LZ03 exhibited a close relationship to phage ZC01 (Fig. 4C).

### Isolated phages are rather stable at different temperatures and pHs.

**FIG 5:**
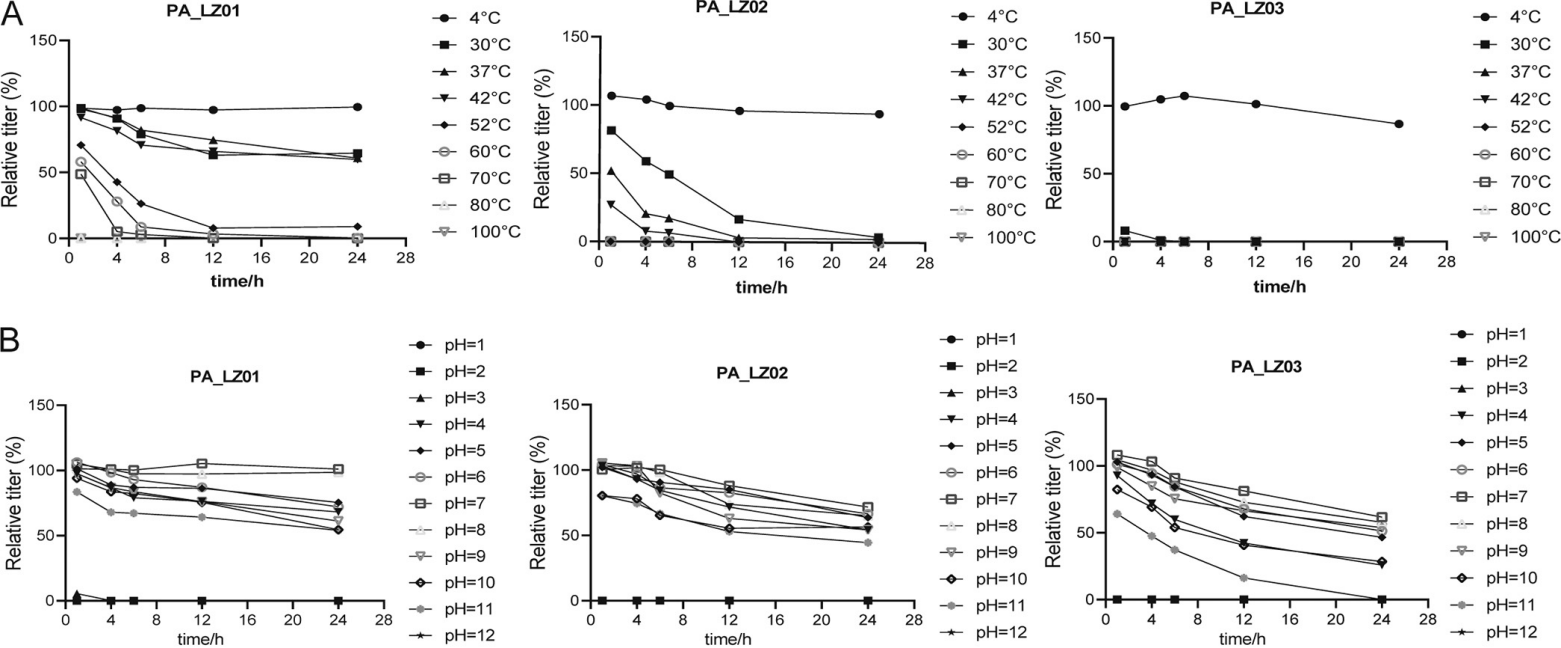
Numbers of infectious phage particles at different temperatures and pHs. (A) PA_LZ01, PA_LZ02, and PA_LZ03 incubated for 1, 4, 6, 12, or 24 h at the indicated temperatures. (B) PA_LZ01, PA_LZ02, and PA_LZ03 incubated for 1, 4, 6, 12, or 24 h at different pHs. Three independent experiments were performed. Error bars represent the SEM.

We further explored the physiological characteristics of these bacteriophages. For temperature stability, the infectivity of the three phages was barely affected when they were incubated at 4°C for 24 h (Fig. 5A). The heat resistance of the three phages was obviously decreased with higher temperatures and longer incubation times. PA_LZ03 completely lost its infectivity at 37°C or higher (Fig. 5A). PA_LZ01 and PA_LZ02 exhibited similar thermal stability patterns and were completely inactivated at 80°C or higher. We found that phage PA_LZ01 possessed good stability within physiological temperature ranges.

Regarding pH stability, all three phages were inactivated when exposed to pH 1, 2, or 12 for 1 h (Fig. 5B). Notably, all viruses maintained their infectivity when exposed to pH 4 to 11, and the pH profile showed that they were most stable at neutral pH (Fig. 5B). Likewise, the pH stability of the three phages was decreased with longer incubation times under acidic or alkaline conditions. PA_LZ01 and PA_LZ02 were more stable than PA_LZ03 at pH 11 (Fig. 5B). Together, these results indicated that PA_LZ01 exhibited the highest stability, while PA_LZ02 was more resistant to alkaline conditions and PA_LZ03 was more resistant to acidic conditions.

### Growth characteristics and lysis kinetics.

**FIG 6:**
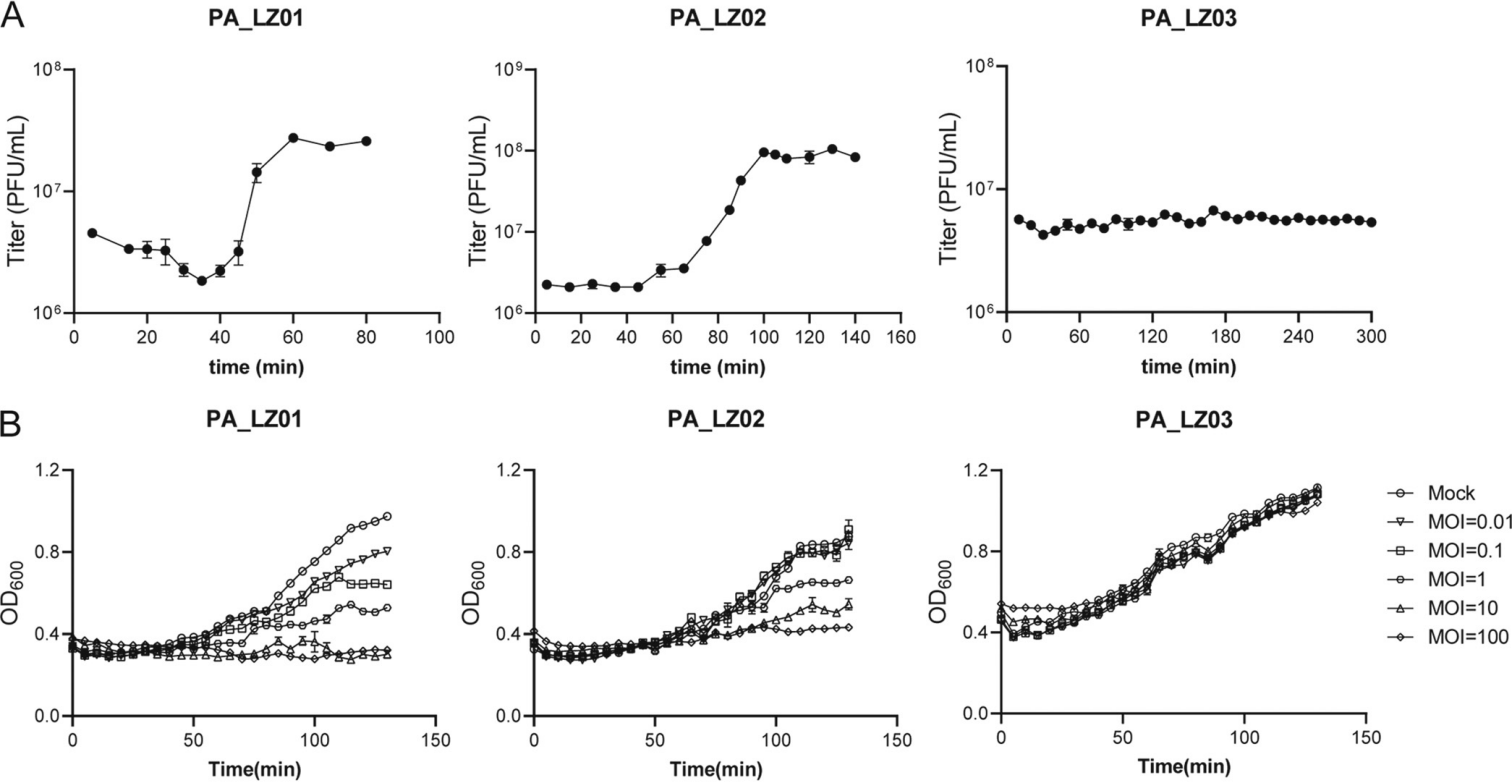
One-step growth curves of different phages and growth curves of P. aeruginosa infected with certain phages. (A) One-step growth curves of phages. The phage titer was calculated at the indicated time points by using a double-layer overlay assay. Three independent experiments were performed. Error bars represent the SEM. (B) P. aeruginosa PAO1 and PA14 strains were mock infected or infected with certain phages at different MOIs (0.01, 0.1, 1, 10, or 100). The growth curves were measured by monitoring the OD_600_. Error bars represent the SEM.

Subsequently, a one-step growth curve was employed to quantitatively describe the proliferation laws of the three phages. PA_LZ01 had an incubation period of about 40 min and a rising period of about 20 min, producing about 15 virion progenies per infected cell (Fig. 6A). In contrast to PA_LZ01, PA_LZ02 had a longer latency period, which was about 60 min, and also had a longer rise period, which was about 50 min (Fig. 6A). However, the burst size of PA_LZ02 was approximately 50 PFU/infected cell, which was higher than that of PA_LZ01 (Fig. 6A). The one-step growth curve of PA_LZ03 was different from those of the other two phages, as its curve remained unchanged and the titer was not greatly increased (Fig. 6A), suggesting that phage PA_LZ03 is a temperate phage.

We further monitored bacterial growth after infection with phages at different multiplicities of infection (MOIs). Host bacteria were cultured to an optical density at 600 nm (OD_600_) of 0.8 and then infected with phages at MOIs from 0.01 to 100. For PA_LZ01 and PA_LZ02, infection at high MOIs (MOIs of 10 and 100) leads to strong bacterial killing with a substantially decreased OD_600_ value (Fig. 6B). When the host bacteria were infected at low MOIs (MOI of 0.01, 0.1, or 1), the growth of the bacteria was not greatly affected. Interestingly, infection with PA_LZ03 did not profoundly affect the growth of the host bacteria (Fig. 6B), which is consistent with the data in Fig. 6A. Together, our results demonstrate that PA_LZ01 and PA_LZ02 have the ability to lyse host bacteria.

### Phage infection reduces P. aeruginosa biofilms.

**FIG 7:**
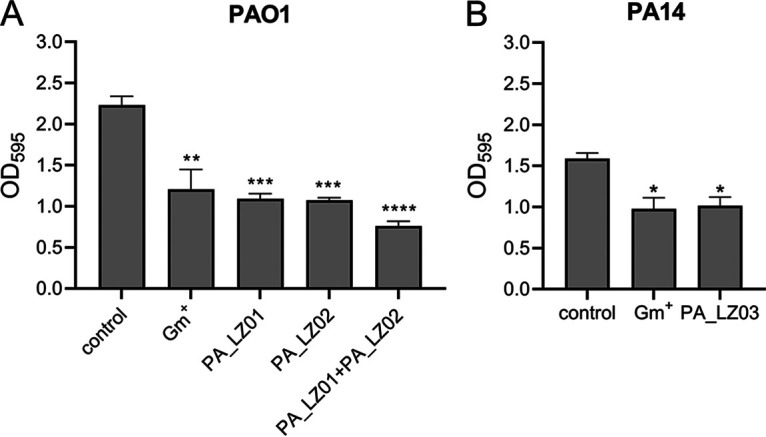
Bacteriophages reduce P. aeruginosa biofilm formation. Biofilm formation was measured by crystal violet staining. Control, without any phage infection; Gm^+^, treatment of biofilms with gentamicin (100 ng/mL); PA_LZ01, treatment with 10^8^ PFU of phage PA_LZ01; PA_LZ02, treatment with 10^8^ PFU of phage PA_LZ02; PA_LZ01+PA_LZ02, treatment with a mixture of PA_LZ01 and PA_LZ02 (0.5 × 10^8^ PFU of each phage); PA_LZ03, treatment with 10^8^ PFU of PA_LZ03. Error bars represent the SEM. *, *P* < 0.05; **, *P* < 0.01; ***, *P* < 0.001; ****, *P* < 0.0001.

In nature, most bacteria can attach to different surfaces and form biofilms (19). One important feature of P. aeruginosa infections is that they are prone to the formation of biofilms, particularly on wounds of burn patients. Once biofilms are formed, the bacteria are difficult to remove (7, 25). Therefore, infection by phages with biofilm degradation abilities showed great therapeutic potential. To explore whether these three phages have the ability to reduce biofilms formed by P. aeruginosa, we used a crystal violet staining assay. Biofilms formed by PAO1 were treated with different phages for 4 h at 37°C. As expected, we found that the biofilms were effectively reduced by either phage used alone as well as by the combination of the two phages PA_LZ01 and PA_LZ02 (Fig. 7A). In particular, PA_LZ01 and PA_LZ02 had the same ability to clear PAO1 biofilms, which could reach 50% at 4 h postinfection. Notably, the biofilm clearance rate reached 65% with coinfection with the two phages, which suggested that the two phages exerted a combined effect on the clearance of biofilms. However, PA_LZ03 exhibited a much lower biofilm clearance ability, with only 36% of the biofilm being degraded after 4 h of treatment (Fig. 7B). Together, our data showed that all three phages can effectively inhibit the formation of biofilms and that PA_LZ01 and PA_LZ02 exhibited stronger effects.

### Phage treatment reduces bacterial loads and inflammatory responses *in vivo*.

**FIG 8:**
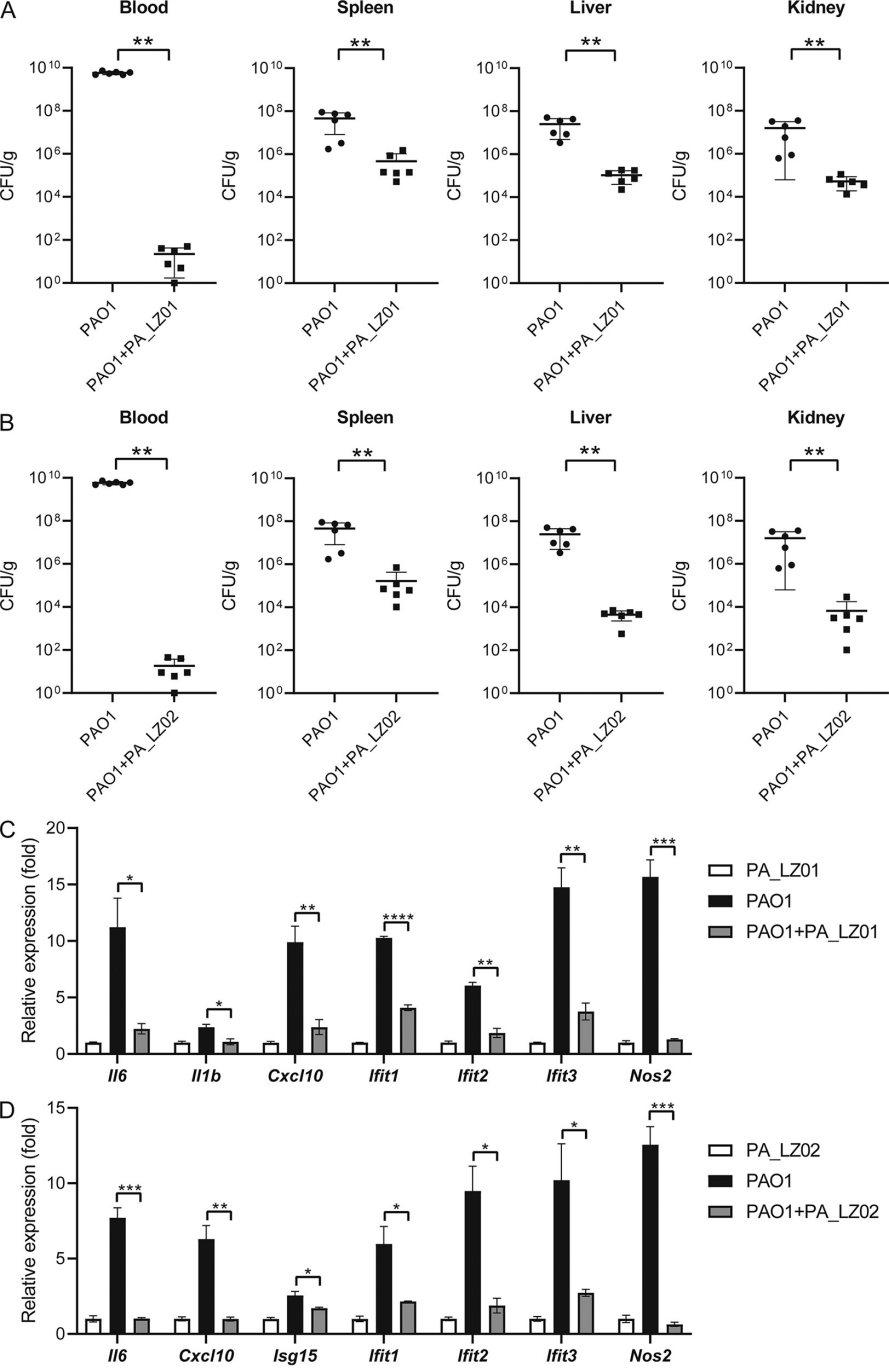
Phage treatment reduces P. aeruginosa loads and inflammatory cytokine expression *in vivo*. (A) Bacterial loads in the spleen, liver, kidney, and blood of C57BL/6 mice that were intraperitoneally infected with the P. aeruginosa PAO1 strain (1 × 10^8^ CFU of PAO1 or 1 × 10^8^ CFU of PAO1 and then 1 × 10^9^ PFU of phage PA_LZ01). (B) Bacterial loads in the spleen, liver, kidney, and blood of C57BL/6 mice that were intraperitoneally infected with the P. aeruginosa PAO1 strain (1 × 10^8^ CFU of PAO1 or 1 × 10^8^ CFU of PAO1 and then 1 × 10^9^ PFU of phage PA_LZ02). Homogenates of different tissues were plated to determine the bacterial CFU counts per gram of organs or per milliliter of blood at 4 h postinfection. (C) Gene expression in the liver of C57BL/6 mice that were intraperitoneally infected with P. aeruginosa (1 × 10^8^ CFU of P. aeruginosa or 1 × 10^8^ CFU of P. aeruginosa and then 1 × 10^9^ PFU of phage PA_LZ01). (D) Gene expression in the liver of C57BL/6 mice that were intraperitoneally infected with P. aeruginosa (1 × 10^8^ CFU of P. aeruginosa or 1 × 10^8^ CFU of P. aeruginosa and then 1 × 10^9^ PFU of phage PA_LZ02). Gene expression was measured by qRT-PCR analysis. The data in panels C and D were normalized to the value for the uninfected control (set as 1). The actin gene was used as the housekeeping gene. Error bars represent the SEM. *, *P < *0.05; **, *P < *0.01; ***, *P < *0.001.

To evaluate the therapeutic effect of phage therapy on P. aeruginosa infection, an intraperitoneal infection mouse model was used. Mice were infected with 1 × 10^8^ CFU of the P. aeruginosa PAO1 strain or 1 × 10^8^ CFU of PAO1 and then 1 × 10^9^ PFU of phage PA_LZ01 or PA_LZ02. As shown in Fig. 8A and B, the blood bacterial load in the PA_LZ01- or PA_LZ02-treated group was significantly lower than that in the P. aeruginosa-infected group at 4 h postinfection. In addition, we further noticed that the numbers of bacteria colonizing the spleens, livers, and kidneys of the phage-treated group were also much lower than those of the P. aeruginosa-infected group (Fig. 8A and B). Infection by P. aeruginosa leads to the potent expression of cytokine genes and interferon-stimulated genes (ISGs) (26). We further explored the expression of inflammatory cytokine genes and ISGs in the spleens and livers of P. aeruginosa-infected or phage-treated mice. As expected, in both the liver and spleen, the expression of inflammatory cytokine genes, including *Il6*, *Il1b*, and *Cxcl10*, and ISGs, including *Ifit1*, *Ifit2*, and *Ifit3*, was induced during P. aeruginosa infection, whereas phage treatment significantly reversed this induction (Fig. 8C and D and Fig. S3). Together, these results showed that treatments with the two lytic phages PA_LZ01 and PA_LZ02 can significantly reduce the bacterial load and inflammatory response in P. aeruginosa-infected mice.

## DISCUSSION

Phage therapy is a promising therapeutic option for patients who are unresponsive to common antimicrobials. The isolation of new phages that can target specific epidemic strains is essential for the clinical application of phages. P. aeruginosa is a leading cause of hospital-acquired infections and exhibits strong natural resistance to antibiotics. Therefore, phage therapy is a viable alternative for the treatment of chronic P. aeruginosa infections, which are a leading cause of hospital-acquired infections and exhibit strong natural resistance to antibiotics (27, 28). In this study, we isolated and identified three phages and two lytic viruses that were effective in clearing P. aeruginosa infections *in vivo*. Treatment with these phages significantly reduced the bacterial loads in the blood and vital organs of a P. aeruginosa-infected mouse model. Moreover, bacterial infection elicited inflammatory responses that were profoundly reduced by phage treatment (Fig. 8; see also Fig. S3 in the supplemental material).

One important challenge in phage therapy is that bacterial resistance can be easily induced due to the strong selective pressure exerted by lytic phages, leading to the rapid selection of phage-resistant bacterial mutants (29). To overcome this, a promising approach is the use of phage cocktails consisting of different strains of phages to treat bacterial infections. In a recent randomized, multicenter, single-blind, a cocktail of 12 phages was used to treat P. aeruginosa infections in burn wound patients (30). While the cocktail was able to reduce the pathogen load in the wounds, it was found to be less effective than the standard treatment of 1% sulfadiazine silver emulsion cream. In our study, we showed that two lytic phages (PA_LZ01 and PA_LZ02) were effective in clearing P. aeruginosa infection *in vivo*. However, we did not compare the effectiveness of combined phage therapy (PA_LZ01 and PA_LZ02) against P. aeruginosa infection. Additionally, the effectiveness of phage therapy and traditional antibiotic treatment against P. aeruginosa infection also requires evaluation.

Biofilm formation is a crucial feature of chronic P. aeruginosa infections. The clearance of bacterial biofilms by phages represents a promising strategy to combat chronic P. aeruginosa infections. Previous studies have isolated phages that can reduce biofilm production to control P. aeruginosa infection (22, 31, 32). The efficacy of phages in reducing biofilm formation has been demonstrated *in vitro* (32, 33). Our study found that PA_LZ01 and PA_LZ02 can effectively reduce P. aeruginosa biofilm formation and that combined treatment with these two phages leads to a strong reduction in biofilm formation (Fig. 7). This reduction is greater than that observed with other P. aeruginosa bacteriophages (24, 34, 35). Although the results are promising, it should be noted that phage-biofilm interactions are complex in the *in vivo* context, and translation of the results from *in vitro* studies to clinical research may not be straightforward. Nevertheless, the possibility of developing phage cocktails using PA_LZ01 and PA_LZ02 is worth considering. Future studies should evaluate the treatment potential of these phages *in vivo* and even in clinical trials. Additionally, efforts should be made to isolate more broad-spectrum phages with different lysis profiles.

The stability of phages is crucial for their clinical application. In this study, we performed various physicochemical experiments to investigate the basic properties of the three isolated phages. We found that PA_LZ01 demonstrated better thermal stability than PA_LZ02, PA_LZ03, and other P. aeruginosa phages such as vB_PaeM_SCUT-S1 and vB_PaeM_SCUT-S2, which were previously isolated by another group (24). The one-step growth curve experiment revealed that the incubation period of PA_LZ01 was approximately 40 min and that the rising period was about 20 min (Fig. 6A), similar to those of vB_PaeM_SCUT-S1 (24) and JG024 (36). When we infected the host bacteria with a high MOI, PA_LZ01 and PA_LZ02 significantly lysed the host bacteria, leading to the inhibition of bacterial growth (Fig. 6B). The inhibitory effect of PA_LZ03 is not strong, which might be due to the fact that PA_LZ03 is a temperate phage.

However, phage therapy is not widely used currently for several reasons that cannot be ignored, the regulatory and safety concerns. One significant challenge facing phage therapy is the regulatory challenges, particularly in countries such as the United States where it is considered an experimental treatment and subject to strict regulations (5). The approval process can be complex, time-consuming, and expensive, which has hindered the development and application of phage therapy. Additionally, there are intellectual-property issues that may retard the development of phage therapy as it can be difficult to patent naturally isolated phages, which can reduce the potential for profits. Phage therapy is also a highly individualized treatment, and the selection of the appropriate phages to target a specific infection requires careful consideration and can be time-consuming, which makes it difficult to establish standardized protocols for treatment (37, 38). Safety concerns are another potential issue, including the potential for allergic reactions or the development of resistance to the phages themselves (39). Despite the risks generally being low, they may still limit the use of phage therapy in some patients or situations. Nevertheless, it is crucial to conduct more research and establish guidelines to ensure the safe and effective use of phage therapy.

Although few phage therapies have been approved for use in human patients, there are many applications of phage therapy such as in food production and veterinary medicine. One application is the use of phage preparations in food production (40, 41). Certain types of phages have been found to be effective in controlling bacterial contamination in food products (42, 43). As a result, phage preparations have been explored for use in controlling bacterial contamination in specific foods such as lunch meats (44). Moreover, phage therapy has been approved for veterinary use in some countries. For example, phage preparations have been used to treat bacterial infections in livestock, such as mastitis in dairy cows (44, 45). However, there may be concerns about the development of resistance to phages over time, and this must be taken into consideration from an environmental protection perspective.

Phage therapy is currently employed in a few different ways, and its preparations typically contain a cocktail of multiple phages that are effective against different bacterial strains (46–48). These phages are selected based on their ability to infect and kill specific types of bacteria, and they are often combined in various ratios to produce a broad-spectrum treatment. Phage preparations used in veterinary medicine may contain a combination of several types of phages that are efficient against different bacterial strains. This approach aims to enhance the effectiveness of treatment by targeting various strains of bacteria simultaneously, thus decreasing the development of antibiotic resistance (49, 50). Phage screening, a process used to identify phages that are effective against bacterial strains, is utilized to create phage cocktails for the treatment of bacterial infections. Through phage screening, different phages are isolated and tested against bacterial strains to identify the most effective ones. Following the identification of a selection of effective phages, they may be combined in various ratios to produce a phage cocktail that is tailored to the specific bacterial strain (47).

Recently, great efforts to develop and refine phage-based treatments for bacterial infections have been made. In addition to the phage therapy cocktails, there are other potential phage-based therapies that have been developed. One area is to the use of nanoparticles to deliver phages directly to infected tissues, thus improving the targeting and effectiveness of phage therapies. Different nanoparticle-based delivery systems, such as electrospun fibers and iron-doped apatite nanoparticles, have been developed to enable precise control over the release and distribution of phages (51, 52). Another approach being studied is the use of engineered phages that have been modified to enhance their effectiveness against bacterial infections (53). For instance, modified phages showed an increased ability to penetrate bacterial biofilms (54). Other modifications may aim to enhance a phage’s stability or its ability to target different bacterial strains or to make it more effective at killing bacteria (55). Taken together, future research should focus on developing and refining phage-based therapies to improve their efficacy and safety for treating bacterial infections.

Here, we isolated and identified three phages (PA_LZ01, PA_LZ02, and PA_LZ03), and their physicochemical properties were extensively investigated. We sequenced three phages and found that phage PA_LZ01 belonged to the genus *Pbunavirus* of the class *Caudoviricetes*, PA_LZ02 belonged to the genus *Pamexvirus* of the class *Caudoviricetes*, and PA_LZ03 belonged to the family *Mesyanzhinovviridae* of the class *Caudoviricetes*. Importantly, we demonstrated that PA_LZ01 and PA_LZ02 are effective in lysing host bacteria and reducing biofilm formation. Furthermore, *in vivo* studies showed that treatment with PA_LZ01 and PA_LZ02 reduced the bacterial loads and inflammatory responses in P. aeruginosa-infected mice. Further research is needed to optimize phage therapy protocols and evaluate their safety and efficacy in clinical settings.

## MATERIALS AND METHODS

### Bacterial strains and growth conditions.

The bacterial strains used in this study are listed in Table S1 in the supplemental material. All bacterial strains were grown in LB broth (1% tryptone, 0.5% yeast extract, 1% NaCl) at 37°C.

### Bacteriophage isolation, propagation, and purification.

Phages PA_LZ01, PA_LZ02, and PA_LZ03 were isolated by using the enrichment method and a double-layer agar overlay to isolate bacteriophages that infect the P. aeruginosa PAO1 or PA14 strain. Untreated wastewater was taken from the First People’s Hospital of Lanzhou City Sewage Center (Qilihe District, Lanzhou City, Gansu Province, China), centrifuged at 5,000 × *g* for 15 min at 4°C, and sterilized with a 0.22-μm filter to remove any endogenous bacteria. One milliliter of the filtered sample and 10 mL of host strains (OD_600_ = 2.0) were added to 100 mL of LB broth in a 250-mL flask and shaken at 37°C at 220 rpm for 24 h. The enrichment culture was centrifuged at 4°C at 5,000 × *g* for 15 min and filtered through a 0.22-μm filter. One hundred microliters of the filtered samples was mixed with 200 μL of host bacteria, and the mixture was incubated for 15 min at room temperature. The mixture was transferred to 5 mL of LB broth with 0.5% agar (top agar), mixed thoroughly, poured over resolidified LB broth with 1.8% agar (bottom agar), and then incubated at room temperature for 30 min before being moved to an incubator and grown at 37°C for 12 to 24 h. Individual plaques were picked by a Pasteur pipette from the LB double-layer agar and resuspended in 1 mL of SM buffer (100 mM NaCl, 8 mM MgSO_4_, and 50 mM Tris-HCl [pH 7.5]). The samples were 10-fold serially diluted with SM buffer, and 100 μL of the diluted samples was taken and mixed with 100 μL of host strains to perform the double-layer agar overlay assay in a 37°C incubator for 12 to 24 h. The above-described steps were repeated to obtain purified phage until all bacteriophage plaques were uniform. The plaque was picked, resuspended in 1 mL of SM buffer, and stored at 4°C until needed. Three different phages were identified by using different host strains (PAO1 and PA14).

Phage propagation was performed by following the steps. One hundred microliters of phage and 1 mL of host strains (OD_600_ = 2.0) were mixed, added to a 1.5-mL Eppendorf (EP) tube, mixed thoroughly, incubated at room temperature for 15 min, poured into 100 mL of LB broth in a 250-mL flask, and shaken at 37°C at 220 rpm for 15 h. The lysate was harvested by centrifugation at 5,000 × *g* for 15 min at 4°C, and the lysate was filtered by using a 0.22-μm filter. The lysate was resuspended with a final concentration of 1 M NaCl, mixed completely, and incubated at 4°C for 1 h. The solution was centrifuged at 5,000 × *g* for 15 min to remove impurities, and the supernatant was then incubated with 10% (wt/vol) polyethylene glycol 8000 (PEG 8000) on ice for 1 h. The mixture was centrifuged again at 12,000 × *g* for 15 min to obtain the phage particles, which were resuspended in SM buffer. The phage solution was incubated with 20% (wt/vol) chloroform for 10 min and then centrifuged at 12,000 × *g* for 15 min to remove PEG 8000, chloroform, and bacterial fragments.

### Transmission electron microscopy.

A total of 3 μL of pure phages was spotted onto carbon-coated grids and allowed to adsorb at room temperature until the drop was dry. Phages were stained with 3 μL 2% potassium phosphotungstate (pH 7) for 1 min and washed 3 to 4 times with H_2_O. The samples were visualized by using a transmission electron microscope (HT7800; Hitachi) operating at 80 kV.

### Temperature and pH stability.

The thermal stability and pH stability of phages were calculated using plaques in the double-layer overlay assay. Briefly, 400 μL of phage (10^6^ PFU/mL for PA_LZ01 and PA_LZ02 and 10^5^ PFU/mL for PA_LZ03) was incubated at different temperatures (4°C, 30°C, 37°C, 42°C, 52°C, 60°C, 70°C, 80°C, or 100°C) for 1, 4, 6, 12, or 24 h, and 400 μL of phage and 400 μL of the host strain were then mixed completely at room temperature for 15 min. The 200-μL mixture was added to 5 mL LB broth with 0.5% agar, dropped onto an LB plate with 1.8% agar, and incubated for 12 to 15 h at 37°C. The thermal stability of the phage was calculated by the number of plaques. The assay of the pH stability of the phages was performed as described above but with changes in the incubation conditions (pH 1 to 12).

### One-step growth curve and lysis kinetics.

A one-step growth curve was performed as follows. One hundred microliters of purified phage and 9.9 mL of host bacteria (OD_600_ = 1.2) were mixed at room temperature for 10 min. The initial phage titer in the mixture was calculated by a double-layer overlay assay. Next, the sample was incubated in a shaker for 180 rpm at 37°C. The samples were collected at 5-min intervals, and the phage titers were determined. The average burst size was quantified as the final and the initial phage titers divided by the initial phage titer. Lysis kinetics were determined as follows. Host strains cultured overnight were diluted 1:100 in liquid LB broth and incubated at 37°C at 220 rpm until the OD_600_ reached 0.8. The host strain was harvested by centrifugation at 5,000 × *g* for 5 min and then washed with liquid LB broth, and the OD_600_ was adjusted to 0.8. One hundred microliters of bacteria and 100 μL of phage were mixed and added to a 96-well plate. The titer of the phage was adjusted accordingly to reach a final multiplicity of infection (MOI) of 0.01, 0.1, 1, 10, or 100. The 96-well plate was incubated at 160 rpm at 37°C, and the OD_600_ was measured every 5 min with a SpectraMax M2 plate reader (Molecular Devices).

### Biofilm clearance assays.

Biofilm clearance was detected by using 96-well plates. Cultures of host strains grown overnight were diluted 1:100 in 100 mL of tryptic soy broth (TSB) medium and incubated at 220 rpm at 37°C until the OD_600_ reached 2.0. Next, bacteria were harvested by centrifugation at 5,000 × *g* for 5 min and washed once with TSB medium. Next, 160 μL of a 100-fold-diluted bacterial solution was mixed, and the mixture was added to a 96-well plate, which was incubated for 24 h at 37°C (TSB was also added to empty wells as a control). The liquid in the 96-well plate was then discarded, and the plate was washed three times with 0.9% NaCl. One hundred microliters of phage or 180 μL of gentamicin (100 ng/mL) was added to the 96-well plate (with 180 μL of TSB as a control), and the plate was incubated at 37°C for 4 h. Next, the liquid was discarded, and the plate was washed twice with 200 μL of 0.9% NaCl. Two hundred microliters of crystal violet was added to the wells for 10 min at room temperature, the crystal violet was discarded, and the plate was washed three times with 220 μL of 0.9% NaCl. Two hundred microliters of acetic acid was added to each well to solubilize the crystal violet. Finally, biofilm formation was quantified by measuring the OD_590_ with a microplate reader.

### Genome extraction and sequencing.

The genomic DNA of the phage was extracted by using a total genomic DNA kit (Cwbio, Jiangsu, China). Purified DNA was used for whole-genome sequencing by Total Genomics Solution Limited. The libraries were constructed with an average length of 350 bp using the NexteraXT DNA library preparation kit (Illumina, San Diego, CA), and the libraries were then sequenced on the Illumina Novaseq 6000 platform. Raw sequence reads were edited using the NGS QC Tool kit (56). High-quality reads were assembled in the phage genome using a *de novo* assembler, SPAdes v3.11.0 (57).

### Genome assembly, annotation, and comparison.

The assembled genomes were annotated by using Prokka, and the sequences were visualized using SnapGene software. The complete genome sequence of the phage was searched for similarity against reported genomes by using BLASTN (https://www.ncbi.nlm.nih.gov/BLAST/). Comparative analysis of the whole genomes was performed by using MAFFT, and the result was visualized by using MEGA. panX was used for the core-gene analysis of phage genomes, and the data were visualized by using iTOL (Interactive Tree of Life) (https://www.embl.de). Genome comparisons were performed by using tBLASTx and visualized by using Easyfig (version 2.2.5) to describe the relationship between the phage and its closest relatives (58).

### Mouse infection.

All mouse experimental procedures were performed according to regulations of the Administration of Affairs Concerning Experimental Animals approved by the State Council of the People’s Republic of China. The protocol was approved by the Animal Welfare and Research Ethics Committee of Northwest A&F University (protocol number NWAFUSM2018001). Six-week-old female mice (C57BL/6) were purchased from the central animal laboratory of Xi’an JiaoTong University (Xi’an, China) and kept under specific-pathogen-free (SPF) conditions. Eighteen mice were equally divided into three groups and injected intraperitoneally with 1 × 10^8^ CFU of P. aeruginosa or 1 × 10^8^ CFU of P. aeruginosa and then 1 × 10^9^ PFU of phage. After 4 h of infection, 100 μL of blood was collected, and bacterial colonies were counted on the plate after serial dilution, which was used to analyze the blood bacterial load. For the analysis of the bacterial loads in the spleen, liver, and kidney, the tissue was taken, weighed, homogenized in phosphate-buffered saline (PBS), serially diluted, spread onto LB plates with 50 μg/mL kanamycin, and incubated at 37°C. In this experiment, parts of the spleen and liver were snap-frozen in liquid nitrogen for subsequent quantitative PCR (qPCR) experiments.

### RNA isolation and quantitative real-time PCR.

C57BL/6 mouse spleen and liver RNAs were isolated using an RNAprep pure tissue kit (catalog number R6201; Tiangen). The purity and concentration of the RNA were determined by gel electrophoresis and spectrophotometry (NanoDrop; Thermo Scientific). RNAs were converted into cDNA with random primers by using a reverse transcription kit (catalog number AH311-02; Transgene). Quantitative real-time PCR (qRT-PCR) was performed by using a SYBR Fast qPCR kit (catalog number KK4601; Kapa Biosystems) on a LightCycler 96 system (Roche), and the data were presented as the mRNA accumulation index (2^−ΔΔ^*^CT^*). The cycling conditions used were as follows: 150 s at 95°C, 45 cycles of 10 s at 94°C, and 30 s at 52°C or 58°C. Data were normalized to the value for the untreated control (set as 1). The actin gene was used as the housekeeping gene. All primers are listed in Table S2.

### Statistical analysis.

Analysis of the significance of the experimental data was performed by using GraphPad Prism 6 (GraphPad Software, San Diego, CA, USA). Statistical analyses for the rest of the assays were performed by using paired two-tailed Student’s *t* test. Error bars represent standard errors of the means (SEM). Significance is indicated by asterisks in the figures (*, *P < *0.05; **, *P < *0.01; ***, *P < *0.001, ****, *P < *0.0001).

### Data availability.

The whole-genome sequences of phage PA_LZ01 were deposited in GenBank under accession number OM953790.1. The whole-genome sequences of phage PA_LZ02 were deposited in GenBank under accession number OQ646789. The whole-genome sequences of phage PA_LZ03 were deposited in GenBank under accession number OQ646790.
