# Supplementary material for: Isolation and Characterization of Three Pseudomonas aeruginosa Viruses with Therapeutic Potential
Source: Microbiol Spectr. 2023 May 1;11(3):e04636-22. doi: 10.1128/spectrum.04636-22 (PMC10269630; doi:10.1128/spectrum.04636-22)
Supplement: Supplemental file 1 — Supplemental material. Download spectrum.04636-22-s0001.pdf, PDF file, 0.3 MB [file spectrum.04636-22-s0001.pdf]

Supplemental Information

Isolation and Characterization of Three *Pseudomonas aeruginosa* Viruses with Therapeutic Potential

Xiao Wang[1,#], Jingjing Tang[1,#], Wen Dang[2,#], Zhen Xie[1], Fuhua Zhang[1], Xinwei Hao[1], Sihuai Sun[1], Xuan Liu[1], Yi Luo[1], Mengyuan Li[1], Yanchao Gu[1], Yao Wang, Qiwei Chen[2]*, Xihui Shen[1]*, Lei Xu[1]*

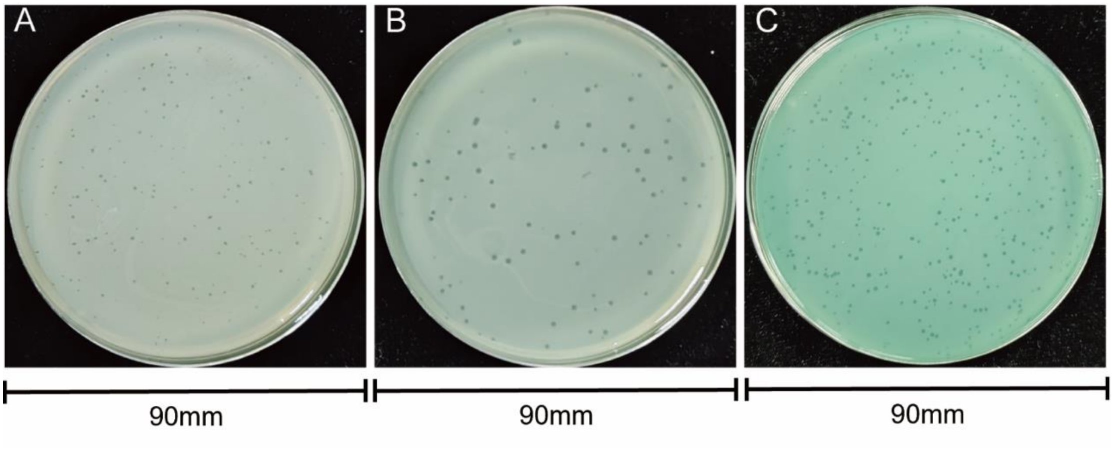

Figure S1. Plaques are formed by different phages.

Plaques formed by phage PA_LZ01 (A) and PA_LZ02 (B) infecting PAO1 and PA_LZ03 (C) infecting PA14.

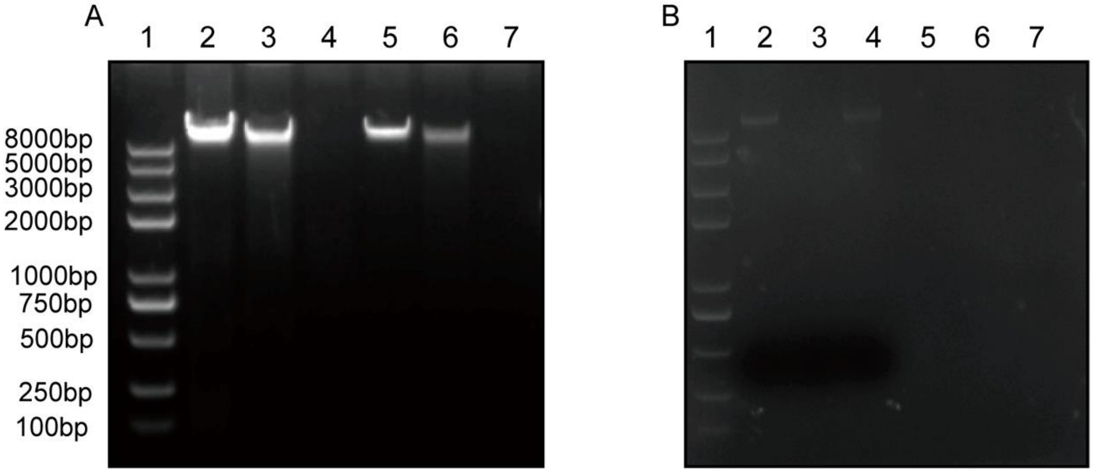

Figure S2. Genomic features of different phages.

(A) Phage PA_LZ01 and PA_LZ02 genomic DNA were digested with DNase I or RNase A, respectively. (B) Phage PA_LZ03 genomic DNA was digested with DNase I or RNase A, respectively.

(A) Lane 1: marker; lane 2: PA_LZ01 genomic DNA; lane 3: PA_LZ01 genomic DNA was digested with RNase A; lane 4: PA_LZ01 genomic DNA was digested with DNase I; lane 5: PA_LZ02 genomic DNA; lane 6: PA_LZ02 genomic DNA was digested with RNase A; lane 7: PA_LZ01 genomic DNA was digested with DNase I.

(B) Lane 1: marker; lane 2: PA_LZ03 genomic DNA; lane 3: PA_LZ03 genomic DNA was digested with DNase I; lane 4: PA_LZ03 genomic DNA was digested with RNase A.

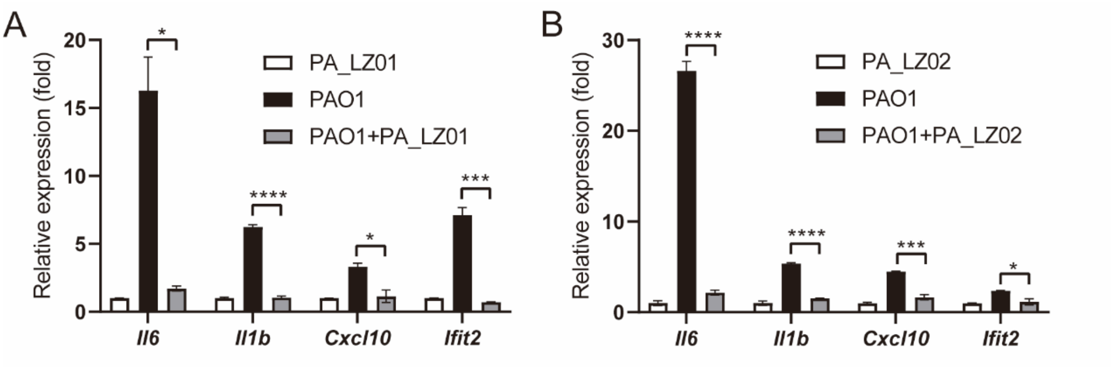

Figure S3. Phage treatment reduces inflammatory cytokines expressions *in vivo*.

(A) Gene expression in the spleen of C57BL/6 mice that were intraperitoneally infected with *P. aeruginosa* strain (1 × 10^8 cfu), or 1 × 10^8 cfu *P. aeruginosa* and then 1 × 10^9 pfu PA_LZ01 phage. (B) Gene expression in the spleen of C57BL/6 mice that were intraperitoneally infected with *P. aeruginosa* strain (1 × 10^8 cfu), or 1 × 10^8 cfu *P. aeruginosa* and then 1 × 10^9 pfu PA_LZ02 phage. Gene expression was measured by qRT-PCR analysis. Data in (A) and (B) were normalized to uninfected control (set as 1). *Actin* was used as the housekeeping gene.

Error bars represent ± SEM. *$P < 0.05$; **$P < 0.01$; ***$P < 0.001$.

Table S1. The bacterial strains and phages used in the study.

Strain	Description
Pseudomonas aeruginosa PAO1	in our laboratory
Pseudomonas aeruginosa PA14	in our laboratory
PA_LZ01	isolated in our laboratory
PA_LZ02	isolated in our laboratory
PA_LZ03	isolated in our laboratory

Table S2. Quantitative PCR primers, related to Figure 8 and Figure S3.

mouse *Actin* F	CATTGCTGACAGGATGCAGAAGG
mouse *Actin* R	TGCTGGAAGGTGGACAGTGAGG
mouse *Isg15* F	CATCCTGGTGAGGAACGAAAGG
mouse *Isg15* R	CTCAGCCAGAACTGGTCTTCGT
mouse *Cxcl10* F	ATCATCCCTGCGAGCCTATCCT
mouse *Cxcl10* R	GACCTTTTTTGGCTAAACGCTTTC
mouse *Ifit1* F	TACAGGCTGGAGTGTGCTGAGA
mouse *Ifit1* R	CTCCACTTTCAGAGCCTTCGCA
mouse *Ifit2* F	CGAACTACCGTCTGGATGACTG
mouse *Ifit2* R	CTTCAACCAGCGCCATTGCTTG
mouse *Ifit3* F	GCTCAGGCTTACGTTGACAAGG
mouse *Ifit3* R	CTTTAGGCGTGTCCATCCTTCC
mouse *Il6* F	TACCACTTCACAAGTCGGAGGC
mouse *Il6* R	CTGCAAGTGCATCATCGTTGTTC
mouse *Nos2* F	GAGACAGGGAAGTCTGAAGCAC
mouse *Nos2* R	CCAGCAGTAGTTGCTCCTCTTC
mouse *Il1b* F	TGGACCTTCCAGGATGAGGACA
mouse *Il1b* R	GTTCATCTCGGAGCCTGTAGTG

Table S3. Host Strains of phage PA_LZ01, PA_LZ02 and PA_LZ03.

	PA_LZ01	PA_LZ02	PA_LZ03
Pseudomonas aeruginosa PAO1	C+++	C+++	C++
Pseudomonas aeruginosa PA14	–	–	C+++

"C+++", each dilution gradient produces clear and transparent plaques, plate shows clear plaques phage ranging in size from 10 to 12 mm. "C++", plaques at 10^2 -10^3 pfu, plate shows clear plaques phage ranging in size from 4 to 6mm. "–", no lysis.

Table S4. The genome annotation of *P. aeruginosa* phage PA_LZ01, PA_LZ02

and PA_LZ03.

Table S5. Comparative genomic analysis of phages that were similar to phage PA_LZ01, PA_LZ02 and PA_LZ03.
